# The Microhardness and Chemical Composition of Different Ceramic Self-Ligating Brackets: An *In Vitro* Study

**DOI:** 10.3390/dj13070285

**Published:** 2025-06-23

**Authors:** Mallaury Martinez, Paul Fawaz, Patrick El Sayegh, Christophe Rapin, Bart Vande Vannet

**Affiliations:** 1 Department of Dentofacial Orthopaedics, Faculty of Odontology, University of Lorraine, 54000 Nancy, France; mallaudentaire@gmail.com; 2 CHRU (Regional University Hospital Center De Nancy), 54000 Nancy, France; 3 Private Practice, Beirut 110720, Lebanon; patrickelsayegh@hotmail.com; 4 Department of Chemistry, Sciences and Technology Faculty, University of Lorraine, 54000 Nancy, France; christophe.rapin@univ-lorraine.fr; 5 Institut Jean Lamour, Artem Campus, Joint Unit of Centre National de la Recherche Scientifique (CNRS) and University of Lorraine, 54011 Nancy, France

**Keywords:** ceramic brackets, chemistry, dentistry, enamel, hardness, orthodontics, orthodontic brackets, SEM, self-ligating

## Abstract

**Objectives:** The aim of this study was to compare the hardness, chemical composition, and microstructure of various self-ligating ceramic orthodontic brackets and enamel. **Methods:** Sixty ceramic brackets (0.022″ × 0.028″) from six different orthodontic firms (Damon^®^ Clear 2, Genius^®^ Crystal, Empower^®^ 2 Clear, Clarity^®^ Ultra, Alpine SL^®^ Clear, and Experience Ceramic^®^) were tested using a microhardness tester and a scanning electron microscope (SEM) equipped with energy-dispersive spectroscopy (EDS). **Results:** The hardness of the ceramic brackets ranged from 1969.8 to 2567.3 VH. The statistical analysis using the Kruskal–Wallis and Mann–Whitney tests revealed significant differences in microhardness between most of the ceramic brackets. Additionally, this study found that passive self-ligating brackets exhibited a significantly higher hardness than that of active self-ligating brackets (*p* = 0.01). The SEM analysis showed that the variations in the oxygen and alumina composition between the six bracket types were also statistically significant (*p* = 0.01). **Conclusions:** Among all of the ceramic brackets tested, Alpine^®^ brackets displayed the lowest hardness values, making them a potential choice for minimizing enamel damage. Notably, the hardness of self-ligating ceramic brackets was found to be at least six times greater than that of enamel, raising concerns about their potential to cause trauma to the enamel of antagonistic teeth. Consequently, the researchers recommend avoiding bonding ceramic brackets to the mandibular teeth or elevating occlusion with turbo-bites to prevent traumatic contact during treatment.

## 1. Introduction

Aesthetic brackets offer an alternative to traditional metal brackets, commonly used in adult patients and some adolescent patients. However, ceramic brackets have distinct mechanical properties, including increased hardness, which may cause wear on the enamel of the opposing teeth [1]. The aesthetic appeal of orthodontic appliances can be ranked in the following order: invisible options (such as lingual braces and clear aligners) demonstrate the highest attractiveness, followed by ceramic brackets, with all-stainless-steel and self-ligating systems rated the lowest [2]. While no significant difference has been found in the treatment efficacy between conventional and self-ligating brackets, there was a significant difference in the time required for a practitioner to place and ligate a 0.014 “NiTi archwire in 6 anterior ceramic brackets” [3].

According to Khan et al., an ideal orthodontic bracket should fulfill several criteria: it must be biocompatible within the oral environment, exhibit long-term stability in both form and function, possess the mechanical properties suitable for the treatment demands, allow for non-iatrogenic placement and removal to protect the dental tissues, effectively transmit forces from the orthodontic archwire, facilitate hygiene maintenance during treatment, be reasonably priced, and satisfy patients’ aesthetic preferences [4]. However, no material currently in use can simultaneously guarantee all of these characteristics.

Hardness is defined as a material’s resistance to penetration by a harder substance and serves as an indicator of its wear resistance. The hardest material will create an impression on the softest [1]. Generally, a significant difference in hardness is undesirable, as it can lead to increased wear. In principle, smaller indentations correspond to higher hardness in the tested material [5]. In our field, we use Vickers microhardness testing, applying loads in the range of a few hundred grams to examine a localized area of the material. These tests are conducted and analyzed under a microscope.

Ideally, the hardness of orthodontic brackets should be less than that of enamel to avoid wear phenomena affecting the opposing teeth [6]. However, the hardness of that bracket should be sufficiently high at the bracket wings to avoid plastic deformation and thus enable the bracket to support and transmit forces generated by the orthodontic archwire that cause tooth movement. The hardness of the bracket’s base should also be lower than that of the wings and the enamel to facilitate bracket removal [7].

The Vickers hardness (VH) of enamel has been studied in many publications. Warkentin et al. found microhardness values ranging from 270 to 420 VH for a maxillary molar [8]. These results are close to those found by Guttierrez-Salazar and Reyes-Gasga, varying between 270 and 360 VH, with the lowest values associated with the cervical area [9]. Similarly, Sa et al. [10] found hardness values for healthy enamel of 316 VH, close to that obtained by Aydin et al. of 330 VH [11], whereas this value was only 39 VH for hypomature enamel with amelogenesis imperfecta. Enamel hardness is also reduced when the enamel is hypomineralized (HME) [12].

Ceramic brackets, due to their greater hardness compared to that of enamel, pose a risk to the enamel of the opposing teeth [13,14]. The Knoop hardness of ceramic brackets is said to be around nine times higher than that of metal brackets (approx. 280 KHN) or enamel (343 KHN) [15]. Only one hardness value has been given in the literature for ceramic brackets since 1988, corresponding to an average value of 1822 VHN for a polycrystalline alumina bar [16].

The hardness of metallic brackets ranges between 350 and 400 VH, and they are found at the wings, leading to a risk of wear of the antagonistic enamel [17]. At the base of the brackets, the hardness may be lower to facilitate their removal at the end of treatment and to be less deleterious to the underlying enamel. This was the case in a study conducted by Eliades et al. on Mini Diamond^®^ brackets (Ormco, Glendale, CA, USA), which found different hardnesses for the base and the wings, at 170 VH and 360 VH, respectively [18]. According to the latest study by Colmant et al., the hardness of metallic self-ligating brackets varies from 203 to 439 VH [19].

After an extensive search of the literature, to our knowledge, no study has yet evaluated the microhardness and chemical composition of different ceramic brackets. Hence, the aim of this study is to compare the microhardness of different ceramic self-ligating brackets and to evaluate their chemical composition.

## 2. Materials and Methods

In this study, six different ceramic brackets 0.022″ × 0.028″ inches in size were chosen (Table 1), to a total sample of *n* = 60. Using the data form a previous pilot study, a sample size of 10 was required to detect a significant difference in the VH between different metallic brackets [19].

− 10 Damon ^®^ Clear 2 brackets (ORMCO, Glendale, CA, USA), lot 062036755;
− 10 Genius^®^ Crystal brackets (Orthopartner, Tainan City, Taiwan, China), lot 5102017112020;
− 10 Empower ^®^ 2 Clear brackets (AO, Sheboygan, WI, USA), lot L68774;
− 10 Clarity ^®^ Ultra brackets (3M, Monrovia, CA, USA), lot LA8HK;
− 10 Alpine SL^®^ Clear brackets (RMO, Denver, CO, USA), lot 20096-43;
− 10 Experience Ceramic^®^ brackets (GC, Breckerfeld, Germany), lot A145931.

Samples of each bracket type were embedded into resin, resulting in twelve strips, each containing five identical brackets. The sequence in which the samples were tested for hardness was randomized. Once a sample was positioned in the hardness tester, all of the brackets were tested on the sample before proceeding to the next one. Each sample consisted of 5 identical brackets, allowing for 5 measurements per bracket, which totaled 25 measurements per sample. In total, 300 hardness measurements were conducted in this study (Table 2).

The Vickers microhardness measurements of the self-ligating ceramic orthodontic brackets were made using a PRESI HZ50-4 automatic microdurometer (PRESI, Eybens, France). The measurements were made in accordance with NF EN ISO 6507-1 (ISO 6507-1: 2023, ISO, Vernier, Geneva, Switserland) [20]. The bracket hardness measurements were carried out on the distal part of the brackets, as this surface is generally larger and flatter. As the composition of the bracket is homogeneous, hardness measurements are representative of the hardness of the surface in contact with the enamel of the antagonistic teeth. In this study, the Vickers indentations were all made using a 500 g load and a contact time of 12 s. Measurements were taken automatically and then checked and modified manually by the operator.

To study the composition of self-ligating ceramic brackets using a SEM (scanning electron microscope), a preliminary sample metallization step is required. This was carried out using the Safematic CCU-010 VH (Valence, France) metallizer–evaporator. Ceramics are non-conductive, so a thin 10 nm layer of carbon was deposited onto the surface of our samples to make them conductive. Measurements were carried out using the JEOL JSM-6010LA SEM (InTouchScope^TM^, Peabody, MA, USA). The chosen pressure was 30 Pascal for evacuating charges through the atmosphere. The samples were placed perpendicular to the electron beam, thanks to the “Tilt” function, which was useful for managing the inclination of the samples. A computer connected to the SEM was used to take the photographs and perform the measurements, thanks to the JSM-6010LA software InTouchScope (InTouchScope^TM^, Peabody, MA, USA).

### Statistical Analysis

In order to compare the Vickers microhardness of the self-ligating ceramic orthodontic brackets, a normality test was carried out. It showed that the microhardness values did not follow a normal distribution.

The level of significance was set at a *p*-value ≤ 0.05 for all statistical analyses. The primary outcome variable in this, study was Vickers hardness (VH). The appropriate alpha error was set at 0.05 and the beta error was set a 0.20. Using data from a previous pilot study, a sample size of 10 was required to detect a significant difference in the VH between different metallic brackets [19]. To compare the VH of the self-ligating orthodontic brackets, two normality tests, Kolmogorov–Smirnov and Shapiro–Wilk, were performed. They showed that the VH values did not follow a normal pattern

As a result, a non-parametric Kruskal–Wallis test for independent samples was carried out to compare the microhardness between the six different self-ligating ceramic orthodontic brackets. The same test was used to compare the oxygen and alumina mass percentages of each sample.

The non-parametric Mann–Whitney test was performed to compare the microhardness of active self-ligating ceramic brackets with that of passive self-ligating ceramic brackets.

Once these statistical tests had been carried out, it was necessary to use a Bonferroni post hoc test to determine significant differences between each group.

A one-sample Wilcoxon rank test was performed to compare the median of each sample with a theoretical median corresponding to the different microhardness values found for enamel, stainless steel, cobalt–chromium, and gold (see Table 3). This test enabled a comparison of

− The average microhardness of each sample to that of enamel;
− The average microhardness of each sample to that of stainless steel;
− The average microhardness of each sample to that of cobalt–chromium;
− The average microhardness of each sample to that of gold.

## 3. Results

**Figure 1:**
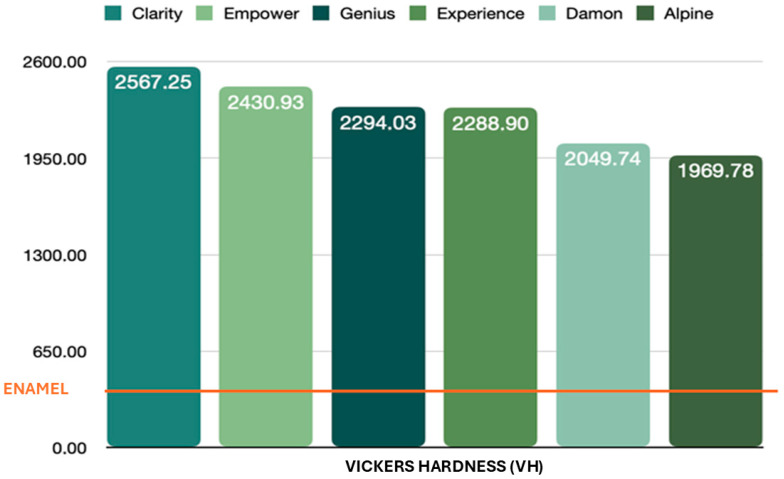
Brackets’ hardness vs. enamel’s hardness.

In this study, the sample comprised 60 brackets, with their Vickers microhardness as the quantitative variable under investigation. Six different self-ligating ceramic brackets were evaluated, with ten brackets of each type tested. Each bracket underwent five measurements, resulting in a total of 50 measurements per bracket type and 300 microhardness measurements overall. Self-ligating ceramic attachments have an average hardness of 2267 ± 60 VH, meaning that self-ligating ceramic brackets are around 6.8 times harder than enamel (Figure 1). The statistical analysis showed a significant difference between the microhardness of the various self-ligating ceramic orthodontic brackets and the microhardness of enamel.

First, the average total microhardness of the self-ligating ceramic orthodontic brackets was compared with that of enamel. Second, the average microhardness of each sample was compared with that of enamel. Calculation of the *p*-value for each sample gave a result of less than 0.001. The statistical analysis showed a significant difference between the different self-ligating ceramic orthodontic brackets (*p*-value = 0.0001).

**Figure 2:**
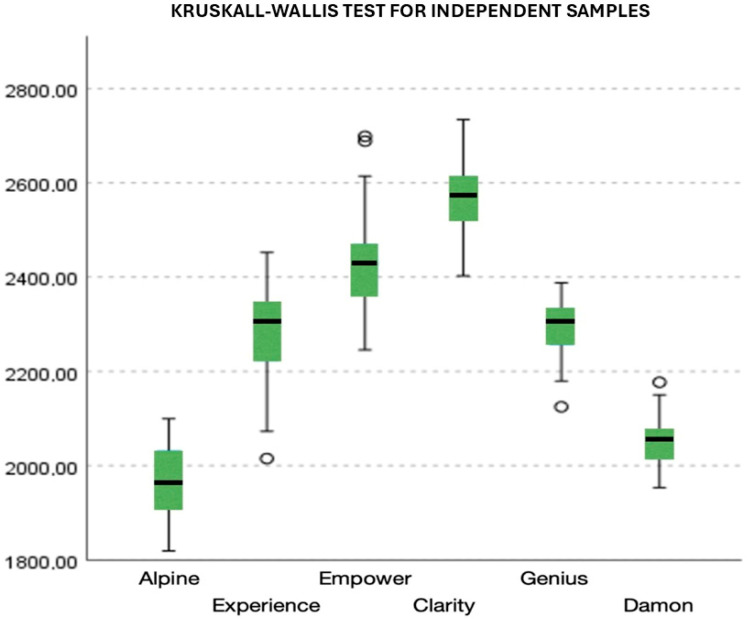
A comparison of the microhardness of different ceramic brackets.

A two-to-two comparison of the different samples showed a significant difference between all groups, except no significant difference was found in the microhardness between the Alpine^®^ and Damon^®^ brackets (*p*-value = 0.097). Also, there was no significant difference in the microhardness between the Experience^®^ and Genius^®^ brackets (*p*-value = 0.983). To a lesser extent, no significant difference in the microhardness between the Empower2^®^ and Clarity^®^ brackets was found (*p*-value = 0.087) (Figure 2).

**Figure 3:**
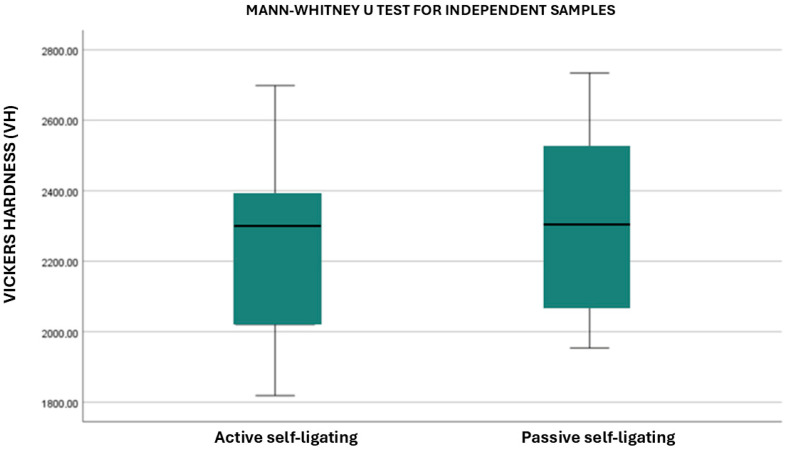
Comparison of the microhardness of different self-ligating systems.

Active self-ligating ceramic brackets (Alpine^®^, Experience^®^, and Empower^®^) were compared to those with a passive system (Clarity^®^, Genius^®^, and Damon^®^). The statistical analysis showed a significant difference between the microhardness of active self-ligating brackets compared to that of passive self-ligating brackets: passive self-ligating brackets had a higher microhardness than those with an active system (*p*-value= 0.01) (Figure 3).

### 3.1. Damon^®^ Clear 2 (ORMCO, Glendora, CA, USA)

The average microhardness of Damon^®^ brackets is 2050 ± 50 VH. The microhardness values found are stable among the 50 measurements carried out on 10 brackets and are higher than those for enamel (331 ± 60 VH). The difference in hardness from that of enamel is significant, with a *p*-value of less than 0.001.

### 3.2. Genius^®^ Crystal (Orthopartner, Tainan, Taiwan)

The average microhardness of a Genius^®^ bracket is 2295 ± 40 VH. The microhardness values found are also stable among the 50 measurements carried out of 10 attachments and are higher than those for enamel. The difference in hardness from that of enamel is significant, with a *p*-value of less than 0.001.

### 3.3. Empower^®^ 2 Clear (American Orthodontics, Sheboygan, WI, USA)

The average microhardness of Empower^®^ 2 was 2431 ± 63 VH, which was significantly different from that of enamel, with a *p*-value of less than 0.001. The microhardness values found were stable among the 50 measurements carried out of 10 attachments.

### 3.4. Clarity^®^ Ultra (3M, Monrovia, LR, USA)

The Clarity^®^ brackets have an average microhardness of 2567 ± 71 VH. The 50 measurements obtained of these 10 attachments are stable. There is a significant difference in hardness from that of enamel, with a *p*-value of less than 0.001.

### 3.5. Alpine SL^®^ Clear (RMO, Denver, CO, USA)

The average microhardness of the Alpine^®^ attachments was 1970 ± 63 VH. The microhardness values found are stable among the 50 measurements carried out of 10 attachments and are significantly different from enamel’s microhardness values. The *p*-value for this difference is less than 0.001.

### 3.6. Experience Ceramic^®^ (GC, Breckerfeld, Germany)

An average microhardness of 2289 ± 72 VH was recorded for the Experience^®^ brackets, with stable values among the 50 measurements carried out of the 10 attachments. Here again, the difference from enamel’s hardness is significant, with a *p*-value of less than 0.001.

**Figure 4:**
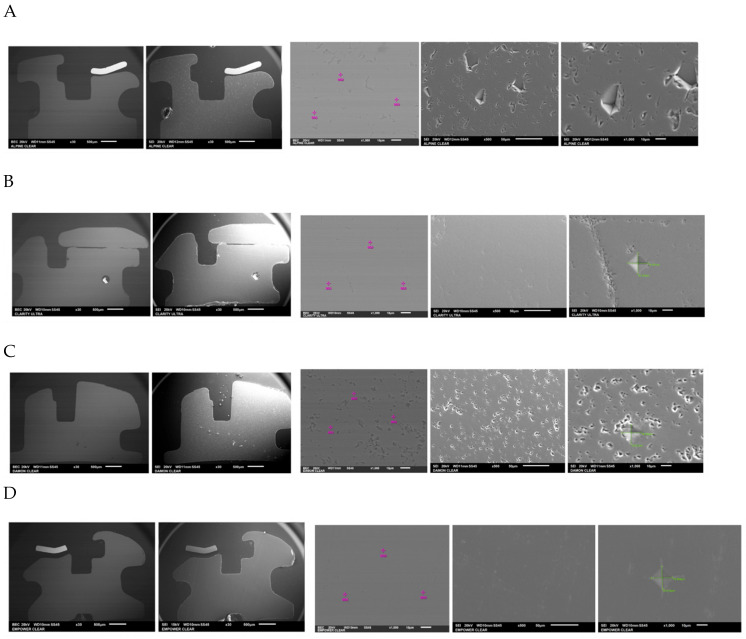

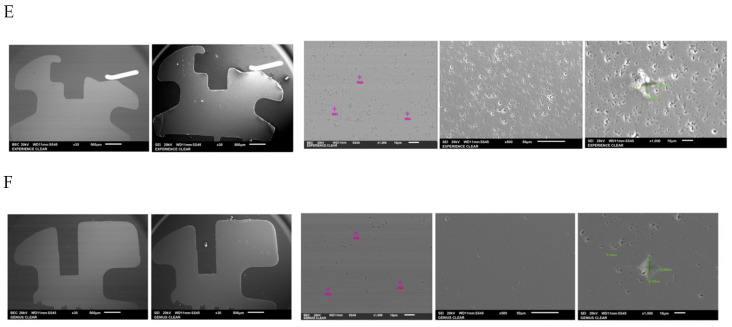
The different bracket types viewed using the SEM (×30/×500) and the locations at which their chemical composition was with visualization of indenter in green. (**A**) Alpine SL^®^ Clear (RMO, Denver, CO, USA). (**B**) Clarity^®^ Ultra (3M, Monrovia, CA, USA). (**C**) Damon^®^ Clear 2 (ORMCO, Glendale, CA, USA). (**D**) Empower^®^ 2 Clear (AO, Sheboygan, WI, USA). (**E**) Experience Ceramic^®^ brackets (GC, Breckerfeld, Germany). (**F**) Genius^®^ Crystal brackets (Orthopartner, Tainan City, Taiwan).

The chemical composition of the orthodontic brackets was studied using a scanning electron microscope (SEM) equipped with an energy-dispersive spectroscopy (EDS) detector (Figure 4). Each sample was tested at three randomly selected locations on the bracket. A composition analysis was carried out on previously metallized samples.

The self-ligating ceramic orthodontic brackets were compared according to their oxygen mass percentages. The statistical analysis showed a significant difference between the six different types of brackets in terms of their oxygen composition (*p*-value = 0.01). Three measurements of the oxygen composition were recorded per bracket type, giving a relatively small sample size (*n* = 3). In the pairwise comparisons, given the small size of the sample (*n* = 3), only the Genius^®^ and Damon^®^ brackets showed significantly different results (*p*-value = 0.009).

The statistical analysis also showed a significant difference between the six different types of brackets in terms of their alumina compositions (*p*-value = 0.01). Three measurements of the alumina composition were recorded per bracket type (*n* = 3). In the pairwise comparisons, with a small sample size (*n* = 3), the Genius^®^ and Damon^®^ brackets also differed significantly, with a *p*-value of 0.009 (Table 4).

## 4. Discussion

The aim of this study was to compare the microhardness of different ceramic self-ligating brackets and to evaluate their chemical compositions. Mandibular incisor brackets were chosen because they cause the most wearing on the incisal edges of the maxillary incisors. The brackets included in the study are both passive and active, allowing for an analysis of all self-ligating systems on the market.

The use of 10 brackets for each type of bracket tested enabled the correct sample power to be obtained. Carrying out 5 measurements per bracket, giving a total of 300 measurements for this study, provided good external validity and allowed the results obtained to be generalized. Only one bracket of each type was tested for its chemical composition. Three measurements were taken at three different points on the bracket to check whether its composition was homogeneous across its entire surface. The number of testers was relatively small but enabled us to identify significant differences between the different bracket types.

For the purposes of this study, all Vickers indentations were made with a spacing of 250 µm between them, a load of 500 g, and a contact time of 12 s. These experimental conditions are similar to those used in previous studies on the hardness of orthodontic brackets [18]. These conditions also made the measurements reproducible. The use of an automatic microdurometer reduced the risk of setup and measurement errors. The measurements were carried out by a single operator. These measurements were repeated several times and at different times to ensure intra-observer reproducibility. The chemical composition study was carried out on the distal surface of each bracket, i.e., not a surface in direct contact with the antagonist teeth. However, this site was chosen because it was large enough for the tests to be performed and was easily identifiable.

As the various attachments were manufactured through ceramic injection molding (CIM = ceramic injection molding), the composition found in this part of the bracket should be identical to that found on the surface in direct contact with the antagonist teeth. The composition was therefore studied at three randomly selected points for greater accuracy of the results [22].

The hardness of a material is generally determined by its chemical composition, internal structure, and manufacturing technique. Self-ligating ceramic orthodontic brackets are manufactured using the same process. By studying the chemical composition of different self-ligating ceramic orthodontic brackets, we are able to understand the microhardness values and explain the difference in microhardness found between different brackets. The microhardness of the self-ligating ceramic orthodontic brackets tested averaged at 2267 ± 60 VH, making them highly traumatic to the enamel of antagonistic teeth. It is significantly different between each type of bracket and in comparison with that of enamel, stainless steel, cobalt–chromium, and gold alloys. Data from the literature on this subject are very weak to non-existent but agree with the values found in this study. The average value for a polycrystalline alumina strip is 1822 VH [16,22].

However, our result differs from the study of Viazis et al., which reported that the Knoop hardness of ceramic attachments is almost nine times higher than that of metal brackets or enamel. This value would lead to brackets with a hardness of around 3000 VH. This value has never been found in laboratory tests [23]. This difference in microhardness between different brackets is explained by the difference in composition and microstructure between them.

The more oxygen there is in the composition of the bracket, the lower its microhardness. However, if we follow this reasoning, the Damon^®^ ceramic brackets should be the least hard, whereas the Alpine^®^ brackets have the lowest microhardness in this study. The manufacturing method, which is not given by every manufacturer, may explain this difference.

The results also show a difference in the microhardness between active and passive self-ligating brackets, but when we look at the microhardness values, which remain very close and very high, we wonder whether this difference really has a clinical impact. The hardness of a material is generally determined by its chemical composition, internal structure, and manufacturing technique.

Studying the chemical composition of the various self-ligating ceramic orthodontic brackets alone cannot explain the difference in microhardness between them. This is probably linked to the conditions under which they were produced (the manufacturing method, grain size and orientation, material microporosity, and sintering temperature) [24,25,26]. However, studying the composition of self-ligating ceramic orthodontic brackets confirms the manufacturers’ claims on the use of alumina oxide. For each of these brackets, the spectra are virtually identical between the three points tested, confirming certain homogeneity in the brackets’ composition.

### Limitations

This study focused on analyzing the microhardness and chemical composition of ceramic brackets, neglecting other factors that may influence bracket performance, such as the bracket design, frictional characteristics, or adhesive properties. This study did not assess the long-term performance of these brackets in clinical settings either, such as their wear resistance or their impact on the teeth over time. Furthermore, the hardness comparison between the brackets and enamel suggests potential damage to the enamel but does not investigate how factors like patient-specific occlusal forces or bracket positioning may influence these outcomes. Lastly, while this research highlights the need to avoid bonding ceramic brackets to the mandibular teeth, it does not provide alternative solutions or explore other methods for mitigating enamel trauma in more complex cases. Future studies are needed to explore these additional factors to provide a more comprehensive understanding of the behavior of ceramic brackets in orthodontic practice. In the future, further studies and clinical trials are needed to also test other important characteristics of these brackets, such as the bonding surface design and their capacity to adhere to dry or contaminated enamel, to complete the overview of the potential overall performance of the brackets [27,28].

## 5. Conclusions

Within the limitations of this study, we may conclude that

o Self-ligating ceramic orthodontic brackets are at least six times harder than enamel and are therefore traumatic to the enamel of the antagonistic teeth. It is advisable to refrain from bonding ceramic attachments to the mandible, or to ensure that the occlusion is elevated with turbo-bites to avoid traumatic contact.
o As the data from the literature on this subject is weak, the present study obtained microhardness values for self-ligating ceramic orthodontic brackets with a satisfactory sample size.
o Studying the brackets’ composition helped explain some of the difference in the microhardness found between the different types of brackets, thanks to their greater or lesser oxygen content.
o Alpine^®^ brackets have the lowest microhardness values and therefore appear to be the brackets of choice among self-ligating ceramic orthodontic brackets.

## Figures and Tables

**Table 1:** Brackets that were studied.

Active Self Ligating Brackets			Passive Self Ligating Brackets		
**Experience Ceramic^®^** (GC, Breckerfeld, Germany) 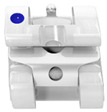	**Alpine SL^®^ Clear** (RMO, Denver, CO, USA) 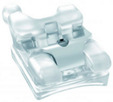	**Empower 2^®^ Clear** (AO, Sheboygan, WI, USA) 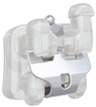	**Clarity Ultra^®^** (3M, Monrovia, CA, USA) 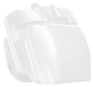	**Genius Crystal^®^** (Orthopartner, Tainan city, Taiwan, China) 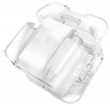	**Damon^®^ Clear2** (ORMCO, Glendale, CA, USA) 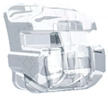
Alumina oxide with PTFE (polytetrafluoroethylene) coating. Clip in cobalt nickel alloy (Co, Ni, Cr, Mo, Fe, Nb, Ti, Mn) with rhodium coating (rhodium, gold).	Polycrystalline ceramic. Clip in nickel titanium with rhodium coating.	Polycrystalline ceramics. Clip: alloy (Co, Ni, Cr, Mo) with rhodium coating.	Polycrystalline ceramics based on very fine microcrystalline silica.	Alumina oxide formula.	Polycrystalline aluminium oxide (PAO) formula

**Table 2:** Sample preparation.

Steps	Protocol	Images
Brackets positioning	The brackets are inserted into a positioning device equipped with 0.19 × 25 stainless steel archwire (identical angulation, perpendicular to the arch segments and presenting the same side to be tested)	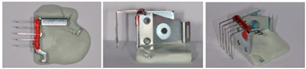
Brackets fixation	Brackets are held in place by sculpting wax (Dentify, Engen, Germany)	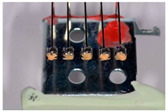
Molding and connecting brackets	Brackets are molded into a flat silicone base using Helio Seal© photopolymerizable compomer resin, to create a flat resin bar that groups brackets from the same series	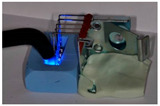
Obtaining sample bars	12 strips are produced, each containing 5 brackets from the same series	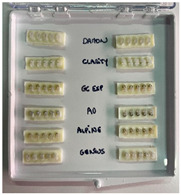
Positioning strips in molds	Bars containing 5 brackets each are fitted with 2 rider clips (Buehler sample support clips) and then placed in molds previously lubricated with an aerosol release agent (Bluesil silicones). 12 molds are made, each containing 1 bar of each bracket	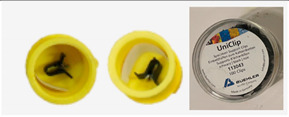
Embedding samples in molds	Samples are coated with ESCIL DBF self-curing araldite epoxy resin, to which ESCIL HY 956 hardener is added. The mixing ratio is 82% monomer to 18% hardener. The components are weighed using a precision balance (KERN PCB, Sigma-Aldrich, St Louis, MO, USA). The resin is reheated to 40 °C to fluidize it and make the monomer/hardener mix more homogeneous.	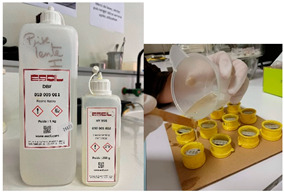
Vacuum packing of samples	The resin is placed for a few minutes under vacuum to remove the bubbles incorporated during preparation	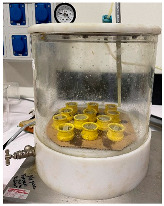
Sample demolding	Once the resin has set completely, the preparations are demolded by hand	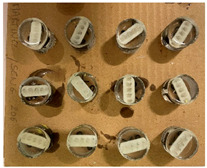
Manual polishing	A first polishing phase is carried out with 2 diamond disks of decreasing granulometry under water. The discs are mounted in a STRUERS ROTOPOL-2 (Struers, Copenhagen, Denmark)manual polisher at a speed of 150 to 300 rpm.	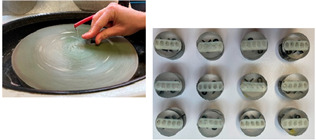
Automatic polishing	A second polishing phase is carried out using cloths loaded with a liquid diamond suspension of equivalent and decreasing particle size (9,6,3,1). The cloths are mounted in a STRUERS TEGRAPOL-31 (Struers, Copenhagen, Denmark) automatic polisher at a speed of 150–300 rpm under a pressure of 20 N/turn for 10/5/5 min	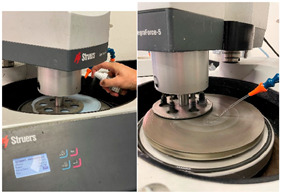
Ready to test samples	The 12 samples are removed from the automatic polisher, rinsed with running water, dried with alcohol and then dried in a hair dryer.	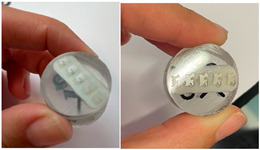

**Table 3:** Microhardness values used in this study based on previous results.

Enamel	Stainless Steel	Cobalt Chrome	Gold
330.52 VH	362.58 VH	335.35 VH	280 VH
Colmant et al. (2023) [19]	Colmant et al. (2023) [19]	Colmant et al. (2023) [19]	Fenjiro et al. (2017) [21]

**Table 4:** Microhardness and chemical composition of different ceramic self-ligating brackets.

		Chemical Composition		Vickers Hardness
		O	Al	(VH)
Alpine^®^	Mean	49.11	50.89	1969.8
	SD	0.6	0.4	62.9
Empower^®^	Mean	48.69	51.31	2430.9
	SD	0.58	0.39	62.7
Genius^®^	Mean	47.97	52.03	2294
	SD	0.6	0.4	40.5
Clarity^®^	Mean	48.87	51.53	2567.3
	SD	0.6	0.4	71.5
Experience^®^	Mean	48.5	51.50	2288.9
	SD	0.59	0.39	72.1
Damon^®^	Mean	50.06	49.94	2049.7
	SD	0.55	0.38	50.2

## Data Availability

The data supporting the findings of this study are available from the corresponding author upon reasonable request.

## References

[1] Papageorgiou S.N., Polychronis G., Panayi N., Zinelis S., Eliades T. (2022). New aesthetic in-house 3D-printed brackets: Proof of concept and fundamental mechanical properties. Prog. Orthod..

[2] Ziuchkovski J.P., Fields H.W., Johnston W.M., Lindsey D.T. (2008). Assessment of perceived orthodontic appliance attractiveness. Am. J. Orthod. Dentofacial. Orthop..

[3] Maizeray R., Wagner D., Lefebvre F., Lévy-Bénichou H., Bolender Y. (2021). Is there any difference between conventional, passive and active self-ligating brackets? A systematic review and network meta-analysis. Int Orthod..

[4] Khan H. (2015). Orthodontic Brackets Selection, Placement and Debonding. J. Orthod. Res..

[5] Darvell B.W. (2018). Materials Science for Dentistry.

[6] Ghafari J. (1992). Problems associated with ceramic brackets suggest limiting use to selected teeth. Angle Orthod..

[7] Kitahara-Céia F.M.F., Mucha J.N., Marques dos Santos P.A. (2008). Assessment of enamel damage after removal of ceramic brackets. Am. J. Orthod. Dentofacial Orthop..

[8] Warkentin M., Freyse C., Specht O., Behrend D., Maletz R., Janda R., Ottl P. (2018). Correlation of ultrasound microscopy and Vickers hardness measurements of human dentin and enamel—A pilot study. Dent. Mater..

[9] Gutiérrez-Salazar M.D.P., Reyes-Gasga J. (2003). Microhardness and chemical composition of human tooth. Mater. Res..

[10] Sa Y., Liang S., Ma X., Lu S., Wang Z., Jiang T., Wang Y. (2014). Compositional, structural and mechanical comparisons of normal enamel and hypomaturation enamel. Acta Biomater..

[11] Aydın B., Pamir T., Baltaci A., Orman M.N., Turk T. (2015). Effect of storage solutions on microhardness of crown enamel and dentin. Eur. J. Dent..

[12] Elhennawy K., Manton D.J., Crombie F., Zaslansky P., Radlanski R.J., Jost-Brinkmann P.G., Schwendicke F. (2017). Structural, mechanical and chemical evaluation of molar-incisor hypomineralization-affected enamel: A systematic review. Arch. Oral. Biol..

[13] Swartz M.L. (1988). Ceramic brackets. J. Clin. Orthod..

[14] Douglass J.B. (1989). Enamel wear caused by ceramic brackets. Am. J. Orthod. Dentofac. Orthop..

[15] Viazis A.D., DeLong R., Bevis R.R., Rudney J.D., Pintado M.R. (1990). Enamel abrasion from ceramic orthodontic brackets under an artificial oral environment. Am. J. Orthod. Dentofac. Orthop..

[16] Zinelis S., Annousaki O., Makou M., Eliades T. (2005). Metallurgical characterization of orthodontic brackets produced by Metal Injection Molding (MIM). Angle Orthod..

[17] Kim K.B. (2018). Orthodontics: Current principles and techniques. Am. J. Orthod. Dentofac. Orthop..

[18] Eliades T., Zinelis S., Eliades G., Athanasiou A.E. (2003). Characterization of as-received, retrieved, and recycled stainless steel brackets. J. Orofac. Orthop..

[19] Colmant M., Fawaz P., Stanton K., MacMichael O., Vande Vannet B. (2023). Microhardness and Chemical Composition of Different Metallic Brackets: An In Vitro Study. Dent. J..

[20] (2023). Metallic materials—Vickers Hardness Test.

[21] Fenjiro I. (2017). Les Attaches Orthodontiques Linguales Ont-Elles Toutes la Même Dureté: Incidences Clliniques. 2025 HAL-05121921v1 (accepted) Mémoire DES ODF 2017.

[22] Zinelis S., Sifakakis I., Katsaros C., Eliades T. (2014). Microstructural and mechanical characterization of contemporary lingual orthodontic brackets. Eur. J. Orthod..

[23] Viazis A.D., DeLong R., Bevis R.R., Douglas W.H., Speidel T.M. (1989). Enamel surface abrasion from ceramic orthodontic brackets: A special case report. Am. J. Orthod. Dentofac. Orthop..

[24] Polychronis G., Papageorgiou S.N., Riollo C.S., Panayi N., Zinelis S., Eliades T. (2023). Fracture toughness and hardness of in-office, 3D-printed ceramic brackets. Orthod. Craniofacial. Res..

[25] Alexopoulou E., Polychronis G., Konstantonis D., Sifakakis I., Zinelis S., Eliades T. (2020). A study of the mechanical properties of as-received and intraorally exposed single-crystal and polycrystalline orthodontic ceramic brackets. Eur. J. Orthod..

[26] Iwasaki T., Nagata S., Ishikawa T., Tanimoto Y. (2022). Mechanical characterization of aesthetic orthodontic brackets by the dynamic indentation method. Dent. Mater. J..

[27] Noorollahian S., Zarei Z., Sadeghalbanaei L., Pakzamir K. (2023). The Effect of Bonding Surface Design on Shear Bond Strength of 3D-Printed Orthodontic Attachments. Int. J. Dent..

[28] Sfondrini M.F., Cacciafesta V., Scribante A., De Angelis M., Klersy C. (2004). Effect of blood contamination on shear bond strength of brackets bonded with conventional and self-etching primers. Am. J. Orthod. Dentofac. Orthop..

